# Red Blood Cells from Individuals with Abdominal Obesity or Metabolic Abnormalities Exhibit Less Deformability upon Entering a Constriction

**DOI:** 10.1371/journal.pone.0156070

**Published:** 2016-06-03

**Authors:** Nancy F. Zeng, Jordan E. Mancuso, Angela M. Zivkovic, Jennifer T. Smilowitz, William D. Ristenpart

**Affiliations:** 1 Department of Chemical Engineering, University of California Davis, Davis, California, 95616, United States of America; 2 Department of Nutrition, University of California Davis, Davis, California, 95616, United States of America; 3 Department of Food Science and Technology, University of California Davis, Davis, California, 95616, United States of America; A*STAR Bioinformatics Institute, SINGAPORE

## Abstract

Abdominal obesity and metabolic syndrome (MS) are multifactorial conditions associated with increased risk of cardiovascular disease and type II diabetes mellitus. Previous work has demonstrated that the hemorheological profile is altered in patients with abdominal obesity and MS, as evidenced for example by increased whole blood viscosity. To date, however, no studies have examined red blood cell (RBC) deformability of blood from individuals with obesity or metabolic abnormalities under typical physiological flow conditions. In this study, we pumped RBCs through a constriction in a microfluidic device and used high speed video to visualize and track the mechanical behavior of ~8,000 RBCs obtained from either healthy individuals (n = 5) or obese participants with metabolic abnormalities (OMA) (n = 4). We demonstrate that the OMA+ cells stretched on average about 25% less than the healthy controls. Furthermore, we examined the effects of ingesting a high-fat meal on RBC mechanical dynamics, and found that the postprandial period has only a weak effect on the stretching dynamics exhibited by OMA+ cells. The results suggest that chronic rigidification of RBCs plays a key role in the increased blood pressure and increased whole blood viscosity observed in OMA individuals and was independent of an acute response triggered by consumption of a high-fat meal.

**Trial Registration:**

ClinicalTrials.gov NCT01803633

## Introduction

Abdominal obesity and metabolic syndrome (MS) are multiplex conditions comprised of several risk factors that increase the future risk of developing diabetes mellitus and cardiovascular disease [1,2,3,4]. As defined by the National Cholesterol Education Program’s Adult Treatment Panel III [5], the diagnostic criteria for metabolic syndrome includes three or more of the following components: abdominal obesity, elevated fasting blood triglycerides and glucose, reduced fasting blood HDL-cholesterol, and high blood pressure. Excess weight gain remains a worldwide problem with 2.1 billion people falling into the overweight/obese category [6]. The incidence of MS has also escalated over the years [7]. According to recently published NHANES data, 34% of the adult US population exhibit MS characteristics [8]. Due to the prevalence of abdominal obesity, MS or other metabolic abnormalities such as insulin resistance and chronic inflammation and associations with increased risk of cardiovascular morbidity and mortality [9,10,11], much work has focused on understanding the contribution of hemorheological alterations to MS [12–19]. The prothrombotic conditions associated with metabolic abnormalities have been shown to increase plasma coagulation, reduce fibrinolysis, decrease endothelial thromboresistance, and cause platelet hyperactivity [20]. Moreover, MS has been associated with increased red blood cell aggregation [15] and microparticles, which come from damaged blood and endothelial cells [21]. All of these factors contribute to the macrorheological profile, and indeed patients with metabolic abnormalities display elevated whole blood, serum, and plasma viscosity compared to healthy individuals [22,23].

One well recognized contributor to whole blood viscosity is the deformability of red blood cells. The ability of RBCs to change shape and deform, especially in the microvasculature, is a main determinant in adequate tissue perfusion [24]. Deformability of RBCs from patients with metabolic abnormalities, however, has not been studied directly; previous work [13,14,16,19] has relied on various indirect techniques. Brun *et al*. [14] and Aloulou *et al*. [16] indirectly calculated the RBC rigidity from measurements of MS+ whole blood viscosity, while Lo Presti *et al*. [13] performed filtration of MS+ whole blood through polycarbonate sieves. In each work they concluded the RBCs were more rigid, but acknowledged that the results may have been influenced by increased plasma viscosity and residual leukocytes and platelets. Likewise, Vaya *et al*. [19] determined erythrocyte deformability using a shear stress diffractometer, which again examines the bulk behavior of many cells. While all of these studies suggest that MS is associated with increased rigidity of RBCs, the measurements were indirect. More importantly, none of the techniques imposed hydrodynamic forces similar to those experienced *in vivo*, i.e., pressure driven flow through a capillary.

Of particular physiological importance is the extensional flow that occurs at the entrance to an abrupt contraction that occurs in cases such as stenoses and aneurysms. Recently, microfluidic devices have been used to elucidate the behavior of RBCs in these physiologically important types of flow [25–32]. In this work, we used a similar microfluidic approach to probe the dynamic behavior of RBCs from individuals with and without OMA as they flow into a constriction to test the hypothesis that OMA+ cells are more rigid than their healthy counterparts. We characterized the behavior (from fasting blood draws) of over 1,156 red blood cells obtained from healthy individuals (n = 5), and 6,668 red blood cells from participants with OMA (n = 4) who were participating in a postprandial (feeding) human clinical trial (the details of which are published elsewhere [33]). We demonstrated that RBCs obtained from participants with OMA exhibited about 25% less deformation on average than healthy RBCs. Furthermore, we investigated the effect of elapsed time during the postprandial period after consumption of a high-fat meal in participants with OMA. Our analysis of 23,000 total RBCs taken from these four participants at 0, 1, 3, and 6 hours postprandial did not show a discernable difference in RBC behaviors or dynamics from fasting samples. These results, along with a careful investigation of the stretching dynamics of 6,668 stretching cells, suggest the decreased deformability is a chronic condition due to the underlying disease state, rather than an acute response triggered by fasting or eating.

## Materials and Methods

### Subjects and Study Design

As part of ongoing studies conducted at the Western Human Nutrition Research Center at University of California Davis, healthy participants and individuals screened for metabolic syndrome (MS) or obesity provided informed consent to participate in this study [33]. Healthy individuals were determined to be healthy based on their answers to a general health history questionnaire that they completed prior to blood collection. Individuals were classified as obese and metabolically abnormal (OMA) if they were obese (BMI ≥ 30) and diagnosed with at least one of the five clinical criteria that define MS: abdominal adiposity (men >102 cm; women >88 cm), fasting circulating triglycerides (≥150 mg/dL), fasting circulating HDL-cholesterol (men < 40 mg/dL; women < 50 mg/dL), fasting blood glucose (≥100 mg/dL) or blood pressure ≥ 130/85 mmHg; or [3] or insulin resistant or were chronically inflamed. Insulin sensitivity was determined from their fasting plasma glucose and insulin using the homoeostasis model assessment of insulin resistance (HOMA-IR) with 2.6 or greater as a cut-off point of insulin resistance [34]. Participants’ inflammation status was determined with the measurement of fasting circulating CRP concentrations at 3mg/L or above as associated with increased cardiovascular disease risk [35]. All participants in the postprandial trial had at least one trait of metabolic syndrome, had a BMI of 30 or greater, and were classified as insulin resistant; and two of the four participants had chronic inflammation (S1 Table). Clinical characteristics of each participant with OMA is found in S1 Table.

In a crossover design, participants with OMA consumed two high-fat dietary test meals after a 10–12 hour overnight fast in random order on two different test days separated by a one to two week washout period. The dietary test meals varied in composition and matrix of fat, as either a cheese sandwich or vegan cheese sandwich, but were equal in total energy, fat, carbohydrates, and protein. The dietary test meals delivered 40% of each individual’s total energy expenditure and provided 50% energy as fat, 40% as carbohydrate, and 10% as protein.

Baseline fasting blood samples were drawn by venipuncture into EDTA tubes prior to the consumption of the test meal, which was to be consumed within 20 minutes. Serial blood draws were taken at baseline (fasting), 1, 3, and 6 hours after consumption of the postprandial challenge. All subjects except one participated in both experimental days. The UC Davis IRB approved all aspects of the clinical study and written informed consent was acquired from each participant; full details of the clinical study are available in reference [33].

### RBC and solution preparation

Blood was collected into EDTA tubes from all participants. The RBCs were processed in a similar manner as presented in [27]. In brief, RBCs were separated from plasma and buffy coat by centrifuging 1 mL of blood at 1200 RPM at 20°C for 4 minutes. The supernatant was removed by aspiration. The packed RBCs were washed three times in physiological salt solution (PSS) buffer, which was made in accordance with the methods described in [27], established by Price *et al*. [36]. The RBCs were diluted with PSS to obtain a 0.5% Hct solution. The low Hct of RBCs was necessary for observations of individual cells in the microchannel. The re-suspended RBC solution was kept at room temperature (~25°C) for at least 30 minutes to ensure constant temperature between samples. All measurements were taken within 12 hours of the blood draw to prevent reduced deformation associated with storage [37].

### Microfluidic platform: fabrication and operation

**Fig 1:**
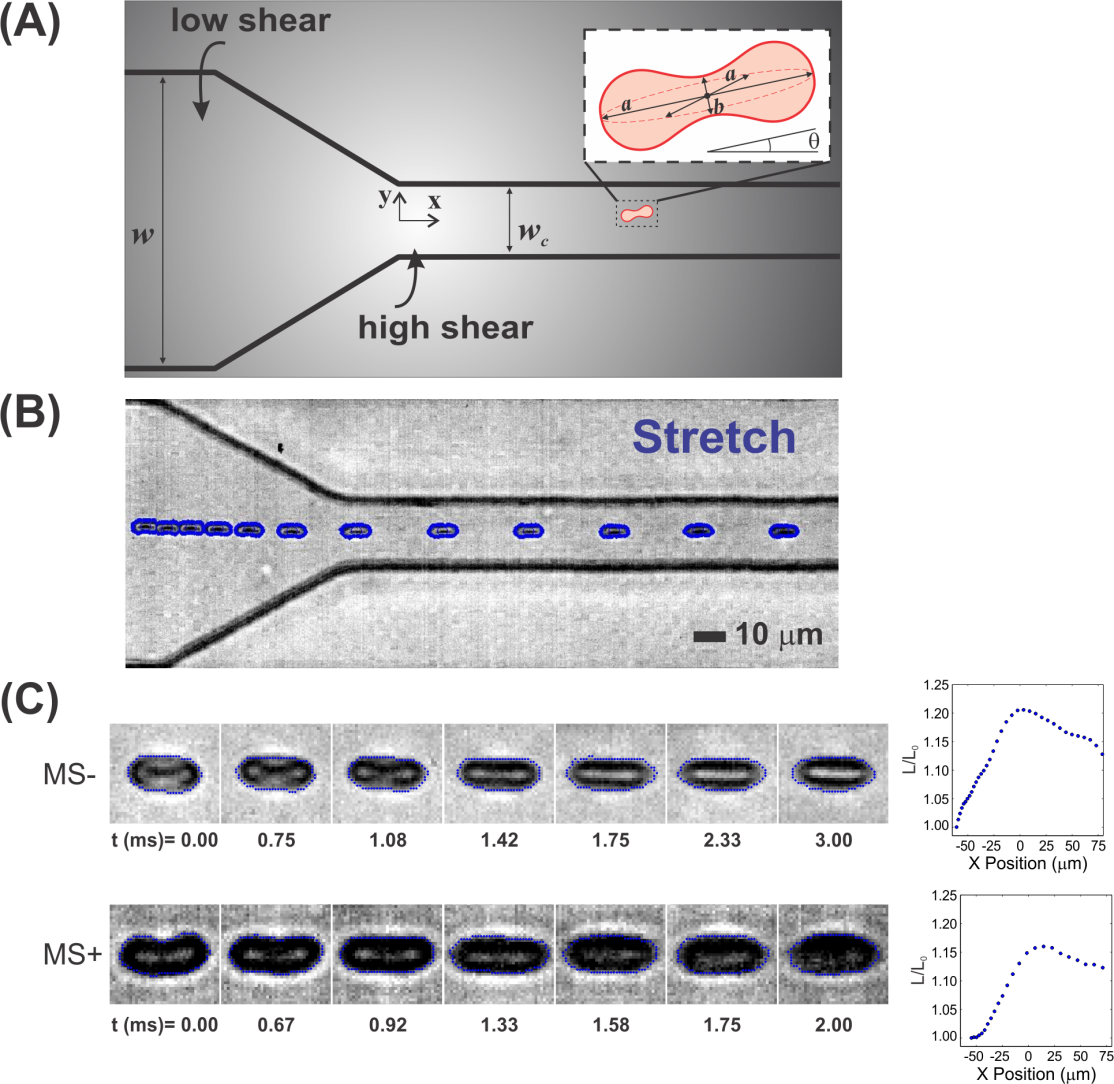
**(A)** Schematic of the microfluidic device. Dilute RBCs are pumped from a region of low shear (a wide channel, at left) to a region of high shear (the constriction, at right). Note that the origin is at the center of the entrance of the constriction as shown on the figure. **(B)** Representative superimposed series of time-lapse images extracted from high speed video each showing an individual RBC entering a constriction and stretching in response to the increased velocity. The original “raw” image of the RBC is shown in grayscale with the detected cell borders superimposed in blue. **(C)** High magnification time lapse images of a representative stretching RBC upon entering a constriction for Control (OMA-) and OMA+. Each image is cropped and centered on the RBC to illustrate its behavior. The box size is 10.2 μm x 10.2 μm. The corresponding plots at the right show major axis length, L, over initial major axis length, L_0_, as a function of the position in the channel.

Our experimental setup and microfluidic device geometry, shown in Fig 1A, is similar to that described in [27]. The microchannels were fabricated with polydimethylsiloxane (PDMS) placed on a glass substrate. The wide sections of the microchannel, before and after the constriction, had a uniform width *w =* 100 μm. The constriction itself had a width *w*_*c*_ = 20 μm and a length of *l*_*c*_ = 800 μm. The channel had a total length of *l* = 1.1 cm and height of *h* = 40 μm.

At the beginning of each experiment, the resuspended solution of 0.5% cells was loaded in a 1 mL syringe. Any visible gas bubbles were then purged and the syringe was connected to a syringe pump to control the flow rate (PHD 2000, Harvard Apparatus). Polyethylene tubes (0.58 mm inner diameter) were used to connect the syringe needle to the inlet hole in the device, and the RBC suspension was pumped through the channel at 5 μL/min. At this flowrate, the average fluid velocity is 2.1 cm/s in the wide region and 10.4 cm/s in the constriction. These corresponding Reynolds numbers ($Re=\frac{\rho \nu L}{\mu }$) are 1.2 and 2.7 respectively, where the characteristic length, L is *h* for the wide region and *w*_*c*_ for the constriction. The microfluidic device has a cross sectional area of 4.0 x 10^−3^ mm^2^ (8.0 x 10^−4^ mm^2^ in the constriction). This produces an average shear rate in the wide region of $\dot{\gamma }\approx \frac{u}{h}\approx \frac{Q}{wh^{2}}\approx 520\;s^{-1}$ where the smallest dimension is the height of the channel. In the constriction region, the width is the smallest dimension and the corresponding shear rate is $\dot{\gamma }\approx \frac{u}{h}\approx \frac{Q}{hw_{c}^{2}}\approx 5200\;s^{-1}$. The extension rate in the taper region, $\frac{\partial u}{\partial x}\approx 1143\;s^{-1}$, is calculated using a taper length of *x* ≈ 70 *μm*. The gradient in the shear rate, $\frac{\partial \dot{\gamma }}{\partial x}$, along the same region is 30 μm^-1^s^-1^. The shear rates experienced in the channel are similar to those found physiologically in arterioles with diameters in the tens of microns range [38].

The RBCs were visualized with a 63x large working distance objective (numerical aperture 0.7) on an optical microscope (Leica). High-speed video of RBC deformations was acquired with a Phantom V7 camera at 12,000 frames per second, with 10–20 μs of exposure time. The center plane was approximated by focusing on the bottom of the slide and moving the stage up to 20 μm. Three separate samples of 0.5% RBCs were prepared for each subject at each time point on each individual day, and three high speed videos were taken at each time point examined post-prandial (either 0, 1, 3, or 6 hours). This methodology yielded a total of 21 individual “trials” for the OMA+ population, where each trial is one high speed video. For the OMA- participants, a total of 14 trials was obtained. A breakdown of the number of cells in each trial is available in S2 Table. All of the resulting movies were analyzed using custom image analysis routines in Matlab, using procedures previously described in detail (see supplementary material of reference [27]), to extract the position, velocity, size and orientation of individual cells, which were used to characterize the RBC motions. As discussed by Zeng & Ristenpart [27], the RBCs typically undergo one of four distinct modes of motion upon entering a constriction: stretching, twisting, tumbling, or rolling. The image analysis procedure identified the type of motion of each individual RBC and extracted quantitative information regarding its dynamics (e.g., the extent of deformation versus time). Our main emphasis here is on the behavior of stretching cells, as characterized by the observed length of the RBC normalized by its initial length, *L*/*L*_0_. We use this parameter rather than the deformation index because RBCs when viewed from the side (as is the case here) have a non-zero DI, even when they are non-deformed (cf. supplementary material of reference [27]). Any cells out of plane were observed to have slight blurring of the cell edges and a change in their intensity, while the blurring may affect image analysis we emphasize that all reported stretching dynamics refer to the normalized stretching parameter *L*/*L*_0_ thus any difference in size caused by location in a different focal plane is effectively cancelled out.

### Statistical Analysis

Statistical analysis was performed using Matlab. Statistical differences were calculated using the Wilcoxon rank sum test, which is appropriate for the non-Gaussian distributions of maximum cell stretching observed here. Differences with p<0.05 were considered to be significant.

## Results & Discussion

### RBC Mechanical Dynamics: Comparison between Metabolic Syndrome and Control

Our experimental procedure yielded quantitative stretching dynamics for a total of 1,156 OMA- individual RBCs and 6,668 OMA+ individual RBCs (cf. S2 Table). Representative time lapse images of RBCs flowing through the microfluidic constriction are shown in Fig 1B and 1C. The RBCs clearly stretch out in response to the accelerating flow, in a manner similar to previous observations [25–30]. After reaching a maximum length, the RBCs also clearly begin to relax, consistent with previous observations using similar microfluidic geometries and flow rates [25–27]. In contrast, other work has seen a plateau in deformation index, stretching, albeit with different geometries and flow rates [28–30]. We emphasize that the majority of cells, at the tested flow rate of 5 μL/min, exhibited mechanical motions other than stretching; all four mechanical motions (stretching, twisting, rolling, and tumbling) were exhibited by both OMA- and OMA+ RBCs. For each high speed video, approximately 20–40% of healthy control RBCs exhibited stretching, while for OMA+ RBCs approximately 20–40% of cells exhibited stretching at a flowrate of 5 μL/min. Likewise, there was no statistically significant difference in the percentage of cells exhibiting the other behaviors (tumbling, rolling, and twisting).

Although the relative fractions of cells exhibiting stretching were comparable for OMA- and OMA+ RBCs, we did observe a quantitative difference in the dynamics of stretching. Here we characterize the stretching dynamics with the ratio of the instantaneous flow-wise length (L) of the RBC to the initial length (L_0_) as a function of X position in the channel. For example, the OMA- cell shown in Fig 1C stretched out to a maximum of L/L_0_ = 1.20, while the OMA+ stretched out only to L/L_0_ = 1.15.

**Fig 2:**
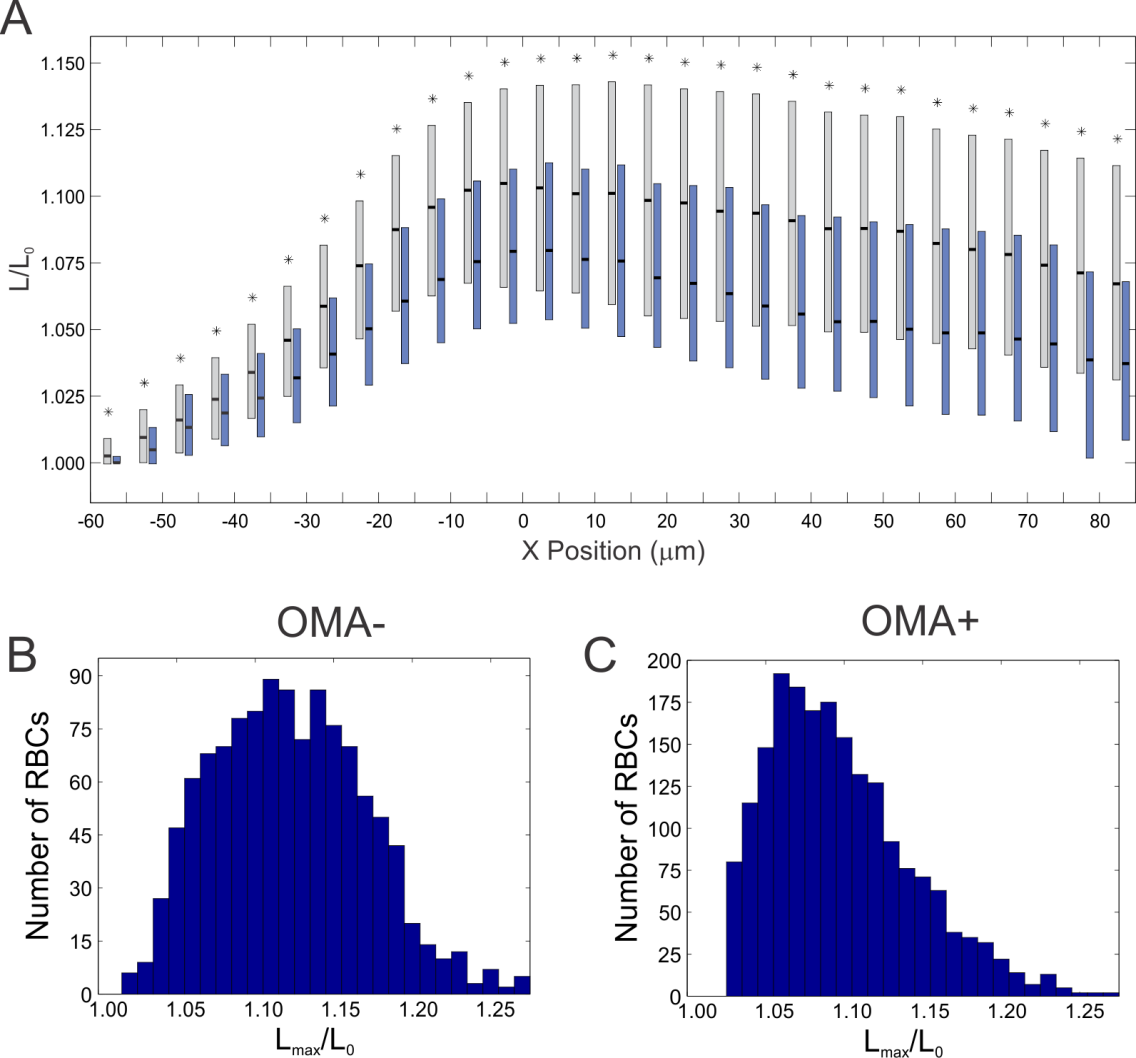
**(A)** Box plot of the ratio of the instantaneous length stretched (L) to initial length (L_0_) as a function of X position in the channel (OMA- = gray boxes, OMA+ = blue boxes). Distributions represent entire population of RBCs examined, from all participants. The top and bottom of the rectangles represent the 25^th^ and 75^th^ quartiles, the thick black bars indicate the median. Bin widths were 5 μm; the boxes for OMA- and OMA+ are staggered within each bin for clarity. Asterisks (*) indicate distributions with p<0.05 compared to the control (OMA-). **(B,C)** Histograms for the maximum length stretched, *L*_*max*_*/L*_*0*_, for **(B)** OMA- RBCs and **(C)** OMA+ RBCs, fasting samples only. Note the distribution is shifted to the left (lower *L*_*max*_) for OMA+ RBCs. The total number of cells observed: n_OMA-_ = 1156, n_OMA+_ = 1955.

Examination of larger populations of RBCs indicates that this discrepancy in stretching magnitude is statistically significant (Fig 2). RBCs from both healthy and participants with OMA stretched in the tapered region of the channel (negative X values), and reached their maximum length near the constriction entrance (X = 0). Interestingly, after entering the constriction and stretching to the maximum length, the RBCs relax as displayed by the gradual reduction in L/L_0,_. The key point in Fig 2A, however, is that OMA+ RBCs tend to stretch less, as indicated by the systematically lower distributions of L/L_0_ for all positions. Histograms of the maximum length stretched (L_max_) normalized by the initial length (L_0_), reveal that the majority of OMA- cells exhibit an L_max_/L_0_ of approximately1.1, the mean value for the population (Fig 2B). For OMA+ RBCs, the shape of the distribution is different, and displays a shift towards lower values, with an average for OMA+ samples of approximately 1.075 (Fig 2C). This reduction in maximum stretch length corresponds to a roughly 25% decrease in the extent of stretching.

**Fig 3:**
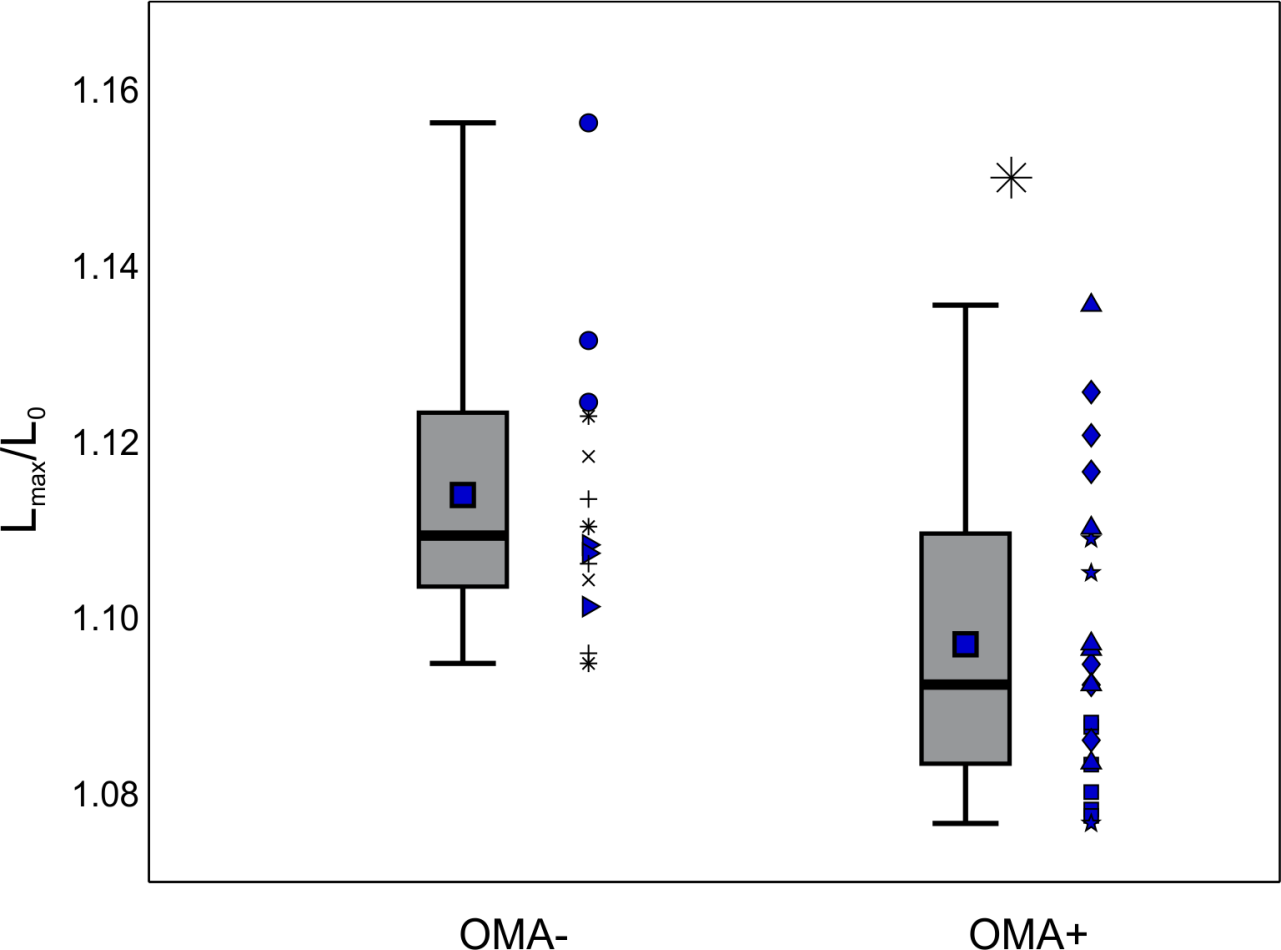
Effect of participant on observed maximum stretching ratio. The top and bottom of the lightly shaded rectangles indicate the 25^th^ and 75^th^ quartiles, respectively, the thick black bars indicate the median, the dark square markers indicate the mean, and the whiskers represent the maximum and minimum values observed. Each marker shape corresponds to a particular individual; repeated markers denote trial replicates with blood drawn from that same participant. All samples are fasting samples only. The total number of cells observed: n_OMA-_ = 1156, n_OMA+_ = 1955. Asterisk (*) indicates p<0.05.

The data shown in Fig 2 represent distributions of thousands of blood cells extracted from different participants, averaged together. This approach obscures any possible variation amongst participants, so to evaluate if there was any participant specific trends we also examined the behavior of RBCs from each participant individually (Fig 3). Here we took the average L_max_/L_0_ of RBCs from each individual for OMA- and OMA+, and plotted the distribution of values as a box plot for each sample. Importantly, although there is overlap in the distributions, the same trend is conserved: L_max_/L_0_ for MS+ exhibits a statistically significant decrease compared to the healthy control (p<0.05).

### RBC Mechanical Dynamics in the Postprandial Period

We investigated the effects of ingesting a meal in patients with metabolic syndrome to assess whether the postprandial period would alter RBC viscoelastic properties and thus the observed mechanical dynamics. In addition, we sought to examine the recovery time after a dietary challenge for RBCs to return to their fasting state. Blood draws were taken at baseline (after fasting 10–12 hours overnight), and at 1, 3, and 6 hours after consuming a meal. The amount of time elapsed postprandial did not have any statistically significant effect on the overall observed RBC behavior percentages (i.e., the fraction of cells that exhibited tumbling, rolling, stretching, or twisting). Likewise, the RBCs isolated from blood draws taken postprandially showed only a weak deviation from the fasting baseline in terms of overall behavior.

**Fig 4:**
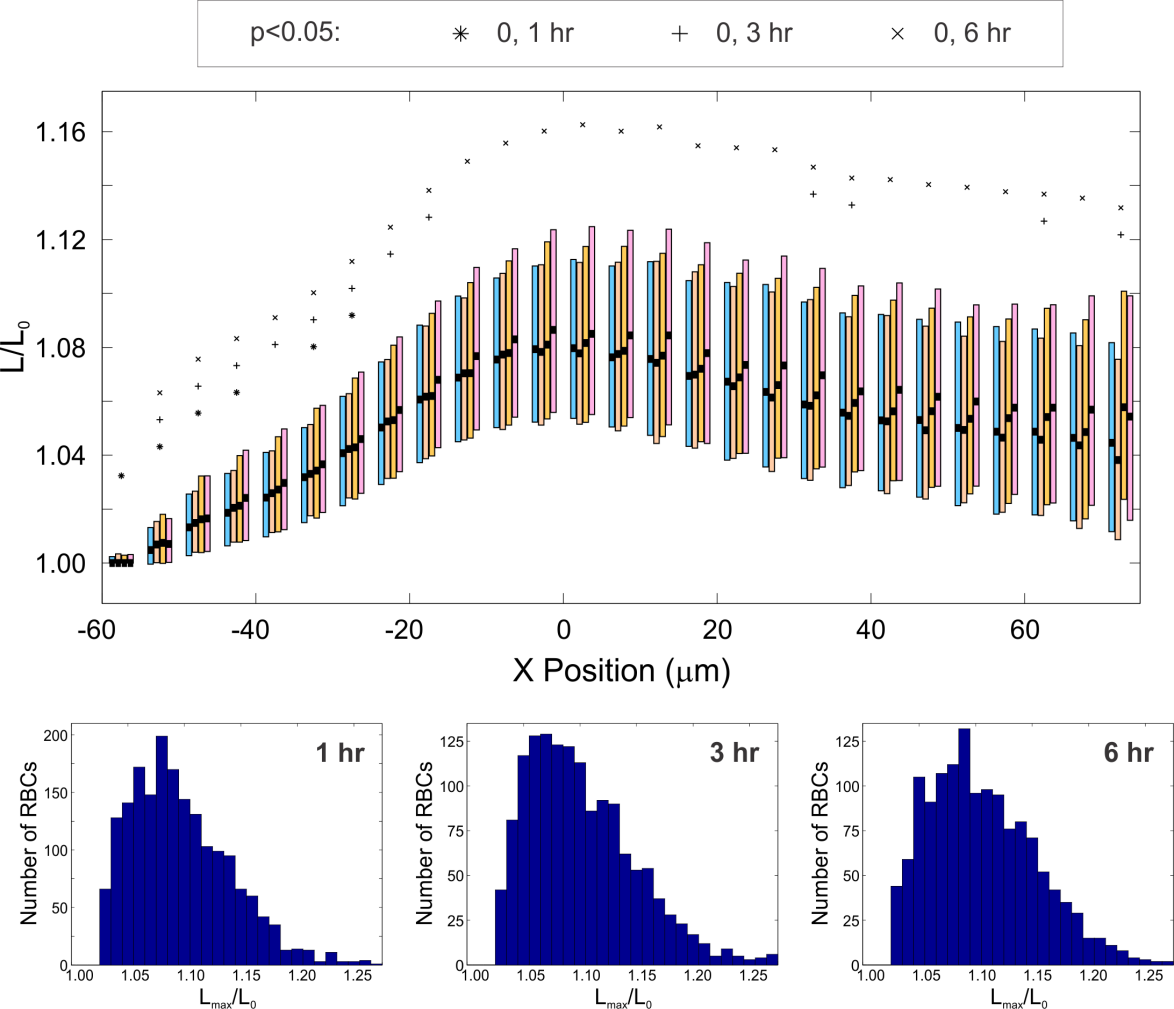
Effect of time elapsed post-prandial on stretching dynamics of OMA+ RBCS. **(A)** Box plots of instantaneous stretching ratio as a function of X position in the channel for blood draws taken postprandially in participants with OMA. The top and bottom of the rectangles represent the 25^th^ and 75^th^ quartiles, the thick black bars indicate the median, and the dark square markers indicate the mean. Shown in the second row are histograms for L_max_/L_0_. The total number of cells observed: n_0HR_ = 1955, n_1HR_ = 1870, n_3HR_ = 1450, and n_6HR_ = 1393

**Fig 5:**
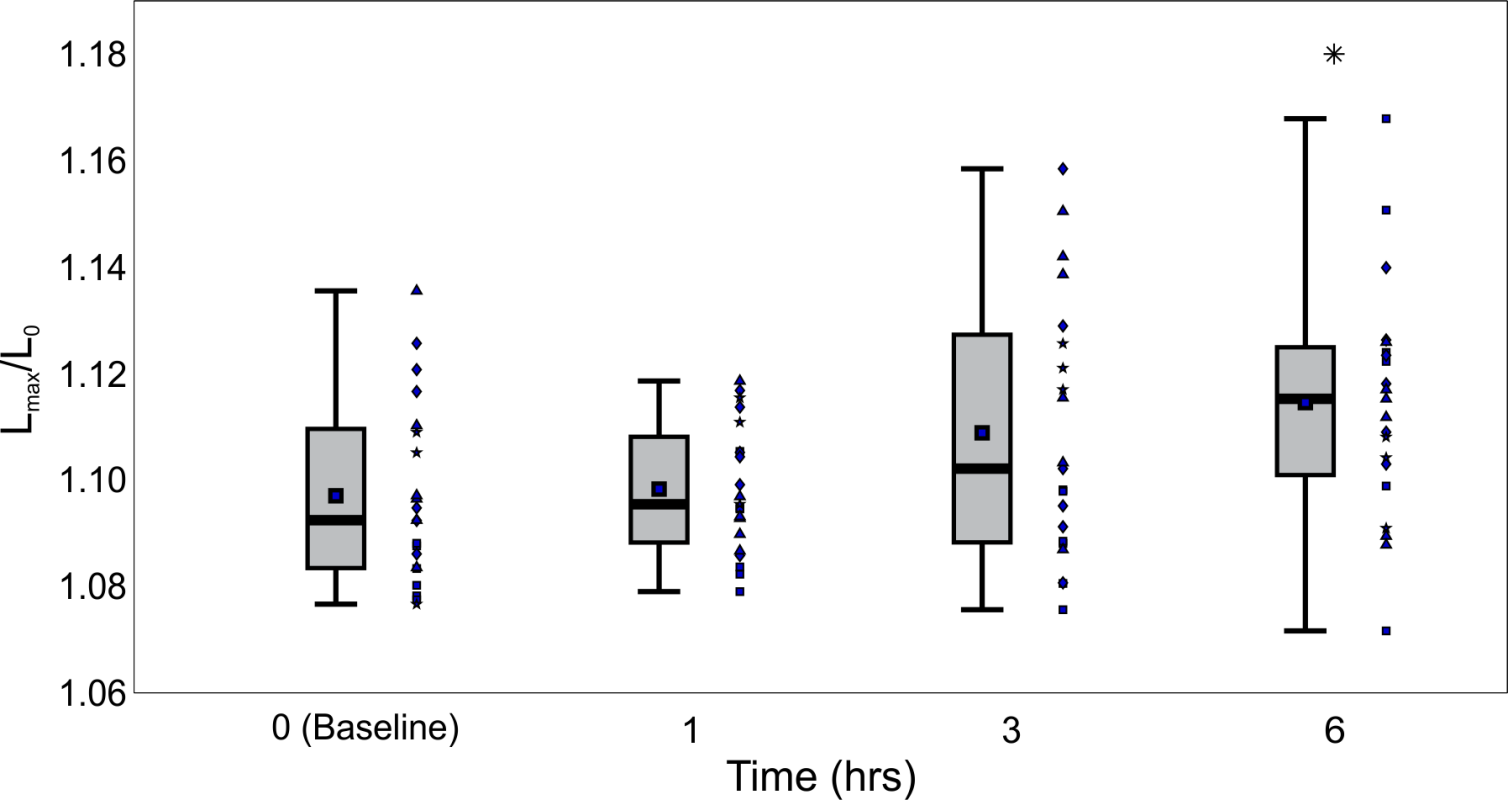
Effect of participant on maximum stretching ratio observed at different times postprandial. The top and bottom of the lightly shaded rectangles indicate the 25^th^ and 75^th^ quartiles, respectively, the thick black bars indicate the median, the dark square markers indicate the mean, and the whiskers represent the maximum and minimum values observed. Each marker shape corresponds to a particular participant; repeated markers denote trial replicates with blood drawn from that same individual. The total number of cells observed: n_0HR_ = 1955, n_1HR_ = 1870, n_3HR_ = 1450, and n_6HR_ = 1393. Asterisk (*) indicates p<0.05.

An investigation of stretching behavior shows that while OMA+ RBCs show statistically significant differences from OMA- RBCs, there is no strong trend over time elapsed postprandially. Fig 4 examines the detailed stretching dynamics showing a boxplot of the instantaneous stretch ratio versus x position in the channel. This plot has staggered boxes showing a side by side comparison of OMA+ RBC behavior for 0, 1, 3, and 6 hours postprandial. Symbols above each box signify statistically significant changes from the baseline. It can be seen from this figure that the only time point postprandial with continuously statistically significant deviations from the fasting baseline is 6 hours, which consistently shows a stretch ratio (L/L_0_) higher than the baseline. This weak effect is consistent with the data in Fig 5, which shows the distribution of maximum stretch distance per participant. Again, the only time point with a statistically significant deviation from the baseline is at 6 hours: the maximum stretch ratio distribution has a higher mean and median than the fasting baseline. These data taken together do not suggest that time elapsed postprandial significantly alters the stretching dynamics, at least over a time period of 1 to 3 hours.

## Conclusion

We have demonstrated that individual RBCs obtained from obese participants with metabolic abnormalities show dynamic stretching behavior in extensional flows that differs significantly from RBCs obtained from healthy individuals. Analysis of the dynamics of stretching motions indicate that OMA+ RBCs stretched on average 25% less at the entrance of a constriction compared to healthy controls. These results corroborate previous work indicating that OMA+ individuals tend to have higher blood viscosity. Our work suggests that one contributor to the increased viscosity is an increased rigidity of individual RBCs. Furthermore, we found little effect of time elapsed postprandial, with OMA+ RBCs showing slight increases in maximum stretch ratio than the fasting baseline. These results directly corroborate the previous indirect indications [13,14,16,19] that OMA+ RBCs are more rigid than healthy RBCs. Taken together, our results suggest that the alterations in RBC mechanical properties associated with metabolic syndrome may have substantial impact on bulk hemorheology.

Metabolic abnormalities include traits that characterize metabolic syndrome and insulin resistance and chronic inflammation [11]. It is composed of a combination of factors, and we emphasize that the results presented here represent the behavior exhibited by RBCs drawn from four OMA+ individuals (albeit with observations on thousands of RBCs drawn from each). A larger scale study is necessary to confirm that decreased RBC deformability is a feature conserved across the population of individuals with OMA. Nonetheless, the results presented here suggest that RBCs from OMA+ participants are chronically rigidified. Because the increased rigidity will have a deleterious impact on the bulk blood viscosity and corresponding blood pressure of participants with OMA, the results presented here indicate the need for further research investigating how increased RBC rigidity contributes to the increased risk of cardiovascular disease and mortality of individuals with OMA.

## Supporting Information

S1 Table Metabolic syndrome (MS) defined by the American Heart Association as waist circumference (WC) > 40 inches for men and 35 inches for women, fasting plasma triglyceride (TG) ≥ 150 mg/dL, fasting plasma high density lipoprotein cholesterol (HDL-C) < 40 mg/dL for men and < 50 mg/dL for women, blood pressure (BP) ≥ 130/85 mmHg, and fasting glucose ≥ 100 mg/dL. (DOCX)

S2 Table Number of cells observed in each trial for OMA+ participants. (DOCX)

S3 Table Number of cells observed in each experimental trial for OMA- participants. (DOCX)
