# Supplementary material for: Red Blood Cells from Individuals with Abdominal Obesity or Metabolic Abnormalities Exhibit Less Deformability upon Entering a Constriction
Source: PLoS One. 2016 Jun 3;11(6):e0156070. doi: 10.1371/journal.pone.0156070 (PMC4892523; doi:10.1371/journal.pone.0156070)
Supplement: S1 Table — (DOCX) [file pone.0156070.s001.docx]

| Partici-pant ID | # MetSyn Traits | Fasting CRP > 3 mg/L | HOMA IR > 2.6 | Gender | Age | BMI | Waist circumference > 35 inches women or > 40 inches men | SBP ≥ 130 mmHg Or DBP ≥ 85 mmHg | Fasting Glucose ≥ 100 mg/dL | HDL-C < 50 mg/dL women or < 40 mg/dL men | Fasting TG ≥ 150 mg/dL |
| --- | --- | --- | --- | --- | --- | --- | --- | --- | --- | --- | --- |
|  |  |  |  |  |  |  |  |  |  |  |  |
| 3006 | 1 |  | X | F | 54 | 30.4 | X |  |  |  |  |
| 3007 | 1 |  | X | F | 53 | 31.6 | X |  |  |  |  |
| 3001 | 2 | X | X | M | 50 | 29.7 |  | X |  | X |  |
| 3016 | 2 | X | X | F | 57 | 35.1 | X |  |  | X |  |

**S1 Table:** Metabolic syndrome (MS) defined by the American Heart Association as waist circumference (WC) > 40 inches for men and 35 inches for women, fasting plasma triglyceride (TG) ≥ 150 mg/dL, fasting plasma high density lipoprotein cholesterol (HDL-C) < 40 mg/dL for men and < 50 mg/dL for women, blood pressure (BP) ≥ 130/85 mmHg, and fasting glucose ≥ 100 mg/dL.
