# Supplementary material for: Red Blood Cells from Individuals with Abdominal Obesity or Metabolic Abnormalities Exhibit Less Deformability upon Entering a Constriction
Source: PLoS One. 2016 Jun 3;11(6):e0156070. doi: 10.1371/journal.pone.0156070 (PMC4892523; doi:10.1371/journal.pone.0156070)
Supplement: S2 Table — (DOCX) [file pone.0156070.s002.docx]

| **OMA+ RBCs** | | | | | | | | | | | | | | |
| --- | --- | --- | --- | --- | --- | --- | --- | --- | --- | --- | --- | --- | --- | --- |
| **Sub** | **Day** | **Time** | **Vid #** | **# cells** | **# str** | **% str** | **Sub** | **Day** | **Time** | **Vid #** | **# cells** | **# str** | **% str** |  |
| 3001 | 1 | 0 | 1 | 356 | 111 | 31.18 | 3001 | 2 | 0 | 1 | 175 | 45 | 25.71 |  |
|  |  |  | 2 | 305 | 92 | 30.16 |  |  |  | 2 | 321 | 99 | 30.84 |  |
|  |  |  | 3 | 320 | 102 | 31.88 |  |  |  | 3 | 266 | 94 | 35.34 |  |
|  |  | 1 | 1 | 249 | 103 | 41.37 |  |  | 1 | 1 | 450 | 117 | 26.00 |  |
|  |  |  | 2 | 367 | 71 | 19.35 |  |  |  | 2 | 272 | 70 | 25.74 |  |
|  |  |  | 3 | 397 | 122 | 30.73 |  |  |  | 3 | 400 | 105 | 26.25 |  |
|  |  | 3 | 1 | 288 | 61 | 21.18 |  |  | 3 | 1 | 196 | 80 | 40.82 |  |
|  |  |  | 2 | 359 | 128 | 35.65 |  |  |  | 2 | 250 | 72 | 28.80 |  |
|  |  |  | 3 | 351 | 95 | 27.07 |  |  |  | 3 | 137 | 29 | 21.17 |  |
|  |  | 6 | 1 | 222 | 45 | 20.27 |  |  | 6 | 1 | 386 | 84 | 21.76 |  |
|  |  |  | 2 | 39 | 5 | 12.82 |  |  |  | 2 | 255 | 69 | 27.06 |  |
|  |  |  | 3 | 56 | 9 | 16.07 |  |  |  | 3 | 282 | 93 | 32.98 |  |
| 3006 | 1 | 0 | 1 | 332 | 98 | 29.52 | 3006 | 2 | 0 | 1 | 305 | 84 | 27.54 |  |
|  |  |  | 2 | 376 | 112 | 29.79 |  |  |  | 2 | 300 | 89 | 29.67 |  |
|  |  |  | 3 | 393 | 129 | 32.82 |  |  |  | 3 | 321 | 103 | 32.09 |  |
|  |  | 1 | 1 | 220 | 71 | 32.27 |  |  | 1 | 1 | 47 | 14 | 29.79 |  |
|  |  |  | 2 | 319 | 84 | 26.33 |  |  |  | 2 | 141 | 28 | 19.86 |  |
|  |  |  | 3 | 344 | 107 | 31.10 |  |  |  | 3 | 219 | 79 | 36.07 |  |
|  |  | 3 | 1 | 240 | 62 | 25.83 |  |  | 3 | 1 | 143 | 6 | 4.20 |  |
|  |  |  | 2 | 514 | 161 | 31.32 |  |  |  | 2 | 165 | 32 | 19.39 |  |
|  |  |  | 3 | 321 | 88 | 27.41 |  |  |  | 3 | 163 | 47 | 28.83 |  |
|  |  | 6 | 1 | 515 | 153 | 29.71 |  |  | 6 | 1 | 124 | 30 | 24.19 |  |
|  |  |  | 2 | 301 | 79 | 26.25 |  |  |  | 2 | 242 | 80 | 33.06 |  |
|  |  |  | 3 | 188 | 40 | 21.28 |  |  |  | 3 | 59 | 5 | 8.47 |  |
| 3007 | 1 | 0 | 1 | 278 | 94 | 33.81 | 3007 | 2 | 0 | 1 | - | - | - |  |
|  |  |  | 2 | 153 | 11 | 7.19 |  |  |  | 2 | - | - | - |  |
|  |  |  | 3 | 416 | 118 | 28.37 |  |  |  | 3 | - | - | - |  |
|  |  | 1 | 1 | 386 | 124 | 32.12 |  |  | 1 | 1 | - | - | - |  |
|  |  |  | 2 | 418 | 146 | 34.93 |  |  |  | 2 | - | - | - |  |
|  |  |  | 3 | 146 | 44 | 30.14 |  |  |  | 3 | - | - | - |  |
|  |  | 3 | 1 | 217 | 81 | 37.33 |  |  | 3 | 1 | - | - | - |  |
|  |  |  | 2 | 171 | 78 | 45.61 |  |  |  | 2 | - | - | - |  |
|  |  |  | 3 | 49 | 22 | 44.90 |  |  |  | 3 | - | - | - |  |
|  |  | 6 | 1 | 208 | 65 | 31.25 |  |  | 6 | 1 | - | - | - |  |
|  |  |  | 2 | 208 | 55 | 26.44 |  |  |  | 2 | - | - | - |  |
|  |  |  | 3 | 201 | 84 | 41.79 |  |  |  | 3 | - | - | - |  |
| 3016 | 1 | 0 | 1 | 224 | 50 | 22.32 | 3016 | 2 | 0 | 1 | 278 | 86 | 30.94 |  |
|  |  |  | 2 | 306 | 80 | 26.14 |  |  |  | 2 | 216 | 67 | 31.02 |  |
|  |  |  | 3 | 341 | 102 | 29.91 |  |  |  | 3 | 523 | 189 | 36.14 |  |
|  |  | 1 | 1 | 396 | 98 | 24.75 |  |  | 1 | 1 | 441 | 147 | 33.33 |  |
|  |  |  | 2 | 228 | 39 | 17.11 |  |  |  | 2 | 401 | 134 | 33.42 |  |
|  |  |  | 3 | 357 | 89 | 24.93 |  |  |  | 3 | 341 | 78 | 22.87 |  |
|  |  | 3 | 1 | 110 | 24 | 21.82 |  |  | 3 | 1 | 222 | 74 | 33.33 |  |
|  |  |  | 2 | 219 | 52 | 23.74 |  |  |  | 2 | 289 | 115 | 39.79 |  |
|  |  |  | 3 | 223 | 61 | 27.35 |  |  |  | 3 | 346 | 82 | 23.70 |  |
|  |  | 6 | 1 | 300 | 111 | 37.00 |  |  | 6 | 1 | 386 | 124 | 32.12 |  |
|  |  |  | 2 | 216 | 57 | 26.39 |  |  |  | 2 | 171 | 73 | 42.69 |  |
|  |  |  | 3 | 382 | 69 | 18.06 |  |  |  | 3 | 344 | 63 | 18.31 |  |
|  |  |  |  |  |  | 31.18 |  |  |  |  |  |  |  |  |
|  | | | | | | | | | | | | | | |
| Total cells | | | | | | | 23102 | | | | | | | |
| Total stretch | | | | | | | 6668 | | | | | | | |

**S2 Table:** Number of cells observed in each trial for OMA+ participants
