# Supplementary material for: Red Blood Cells from Individuals with Abdominal Obesity or Metabolic Abnormalities Exhibit Less Deformability upon Entering a Constriction
Source: PLoS One. 2016 Jun 3;11(6):e0156070. doi: 10.1371/journal.pone.0156070 (PMC4892523; doi:10.1371/journal.pone.0156070)
Supplement: S3 Table — (DOCX) [file pone.0156070.s003.docx]

| **OMA- RBCs** | | | | | | | | | | | | | | |
| --- | --- | --- | --- | --- | --- | --- | --- | --- | --- | --- | --- | --- | --- | --- |
| **Sub** | **Day** | **Time** | **Vid #** | **# cells** | **# str** | **% str** | **Sub** | **Day** | **Time** | **Vid #** | **# cells** | **# str** | **% str** |  |
| 1 | 1 | 0 | 1 | 384 | 101 | 26.30 | 4 | 1 | 0 | 1 | 202 | 37 | 18.32 |  |
|  |  |  | 2 | 442 | 63 | 14.25 |  |  |  | 2 | 300 | 55 | 18.33 |  |
|  |  |  | 3 | 436 | 80 | 18.35 |  |  |  | 3 | 277 | 82 | 29.60 |  |
| 2 | 1 | 0 | 1 | 408 | 57 | 13.97 | 5 | 1 | 0 | 1 | 183 | 34 | 18.58 |  |
|  |  |  | 2 | 362 | 76 | 20.99 |  |  |  | 2 | 165 | 31 | 18.79 |  |
|  |  |  | 3 | 106 | 57 | 53.77 |  |  |  | 3 | - | - | - |  |
| 3 | 1 | 0 | 1 | 255 | 52 | 20.39 |  |  |  |  |  |  |  |  |
|  |  |  | 2 | 365 | 122 | 33.42 |  |  |  |  |  |  |  |  |
|  |  |  | 3 | 548 | 309 | 56.39 |  |  |  |  |  |  |  |  |
|  | | | | | | | | | | | | | | |
| Total cells | | | | | | | 4433 | | | | | | | |
| Total stretch | | | | | | | 1156 | | | | | | | |

**S3 Table:** Number of cells observed in each experimental trial for OMA- participants
